# Effect of Wastewater Treatment on Bacterial Community, Antibiotic-Resistant Bacteria and Endoparasites

**DOI:** 10.3390/ijerph19052750

**Published:** 2022-02-26

**Authors:** Ingrid Papajová, Júlia Šmigová, Gabriela Gregová, Jindřich Šoltys, Ján Venglovský, Ján Papaj, Tatiana Szabóová, Nikola Dančová, Lukáš Ihnacik, Ingrid Schusterová, Jana Sušinková, Jana Raková, Ivana Regecová

**Affiliations:** 1 Institute of Parasitology, Slovak Academy of Sciences, 040 01 Košice, Slovakia; bystrianska@saske.sk (J.Š.); soltys@saske.sk (J.Š.); ihnacik@saske.sk (L.I.); 2 Department of Public Veterinary Medicine and Animal Welfare, University of Veterinary Medicine and Pharmacy in Košice, 041 81 Košice, Slovakia; gabriela.gregova@uvlf.sk (G.G.); jan.venglovsky@uvlf.sk (J.V.); tatiana.szaboova@uvlf.sk (T.S.); nikola.dancova@student.uvlf.sk (N.D.); 3 Faculty of Electrical Engineering and Informatics, Technical University in Košice, 042 00 Košice, Slovakia; jan.papaj@tuke.sk; 4 1st Department of Cardiology, East Slovak Institute of Cardiovascular Diseases, A Joint-Stock Company St., 040 11 Košice, Slovakia; schusterovai@gmail.com; 5 Faculty of Medicine, Pavol Jozef Šafárik University in Košice, 040 11 Košice, Slovakia; jana.susinkov@upjs.sk (J.S.); jana.rakova@upjs.sk (J.R.); 6 Department of Food Hygiene, Technology and Safety, University of Veterinary Medicine and Pharmacy in Košice, 041 81 Košice, Slovakia; ivana.regecova@uvlf.sk

**Keywords:** wastewater, antimicrobial resistance, *E. coli*, protozoan cysts, helminth eggs, sanitation

## Abstract

Wastewater and wastewater treatment plants serve as urban reservoirs of pathogenic microorganisms. Wastewaters frequently contain bacteria, antibiotic-resistant bacteria, and developmental stages of parasites with significant zoonotic potential. Five wastewater treatment plants in the central part of Slovakia were investigated to determine the effect of treatment on bacterial community, antibiotic-resistant bacteria, and the occurrence of helminth eggs. Although all monitored chemical factors (chemical oxygen demand, biochemical oxygen demand, N-NH4, total nitrogen, and total phosphorus) in the effluent were in line with the legislative standards for discharge into public waterways, the results of minimal inhibitory concentrations show that reclaimed water harbors *E. coli* resistant to several commonly used antibiotics (ampicillin, piperacillin, and tazobactam, combine ampicillin and sulbactam, cefotaxime, tetracycline). The presence of endoparasite developmental stages in wastewater and sludge (*Ascaris* spp., *Hymenolepis nana*, eggs from the Ancylostomatidae family, *Giardia duodenalis*) indicates potential health risks for humans and workers at these sites. Treatment such as composting before applying sludge to land is necessary to reduce human pathogens.

## 1. Introduction

Wastewater and wastewater treatment plants serve as urban reservoirs for pathogenic microorganisms [1,2]. Wastewaters frequently contain developmental stages of parasites with likely zoonotic potential. Many viruses and bacteria are devitalized during the treatment process in a wastewater treatment plant, but sometimes not entirely [3,4].

Frequent use of antibiotics in human and veterinary medicine leads to the spread of antibiotic-resistant bacteria into the environment. Antibiotic-resistant strains enter the environment through human feces and the liquid manure of animals. Antibiotics consumption and the increase of microorganisms’ resistance can be seen by higher concentrations in wastewater, agriculture, livestock farming, and the human population [5,6,7]. Antibiotic-resistant bacteria and their genes are significantly emerging environmental pollutants. The wastewater and sludge can be transmitted to agricultural land via agricultural run-off into the aquatic ecosystem and poses the risk of transmission into the food chain [8]. The prevalence of antibiotics in municipal wastewater and surface waters can lead to the development of antibiotic-resistant bacteria due to long-term exposure to low concentrations of antibiotics in the ng/L to μg/L range [9].

*E. coli* is typically chosen as the representative indicator of antimicrobial resistance in Gram-negative bacteria and is responsible for infections in humans and animals [10,11]. As part of the endogenous microbiota, *E. coli* can easily acquire resistance against antimicrobials consumed by humans and animals [12,13].

Antibiotic resistance is primarily caused by antibiotic use, which has led to initiatives to restrict antibiotic prescriptions and curtail antibiotic use in agriculture. Despite this knowledge, antibiotics are still the most commonly used drugs in Slovakia. According to the National Action Plan on Antimicrobial Resistance to antibiotics for outpatients in the Slovak Republic, for the period 2019–2021, antibiotic consumption was slightly higher than in other EU countries [14]. Antibiotics use in healthcare facilities is at the EU level. The significant problem is the resistance of Gram-negative microorganisms such as enterobacteria, pseudomonads, and acinetobacter to the third and higher generation of cephalosporins, fluorinated quinolone, and aminoglycosides. In Slovakia, the immediate and specific problem is the increasing resistance of enterobacteria to carbapenems. Current data on resistance in the Slovak Republic (national surveillance) are available on the website of the Public Health Authority of the Slovak Republic [15]. Furthermore, it is known that helminths cause the most parasitic infections in humans and animals among all pathogenic intestinal parasites. Helminths pose severe health risks due to their lower levels of bacteria, thus enabling very high egg survival rates and inducing resistance to common disinfectants and ultraviolet irradiation [16,17].

Helminths are also often considered as the primary constraint for the reuse of wastewater in agriculture because of their low infective dose and prolonged survival rate in the environment [18,19,20]. Developmental stages of endoparasites have been found in raw wastewater [20]. Wastewaters frequently contain developmental stages of parasites with zoonotic potential (e.g., *Cryptosporidium* spp., *Giardia* spp., *Toxocara* spp., *Echinococus * spp.) [19]. It is reported that 1% of the healthy human population eliminates pathogenic agents who pass with wastewaters to wastewater treatment plants. The most prevalent in the influent segment in different treatment plants are protozoan (oo)cysts *Giardia* spp., *Cryptosporidium* spp., and *Trichostrongylus* spp., *Ascaris lumbricoides*, *Enterobius vermicularis*, *Trichuris trichiura*, and *Hymenolepis* spp. [21,22,23,24,25,26,27,28]. Many factors influence the amount: the population density, the rates of transmission, the economic status of the society, geographical regions, and climatic conditions [29,30,31].

The most important are eggs soil-transmitted helminths (STH). According to the World Health Organization [19], the upper limits for the STH eggs in sludge are 0.25 and 1 helminth egg/g total solids (TS), respectively. Feachem et al. [32] reported a high prevalence of STH eggs in fecal sludge in developing countries (67–735 eggs/g TS) and a lower prevalence in developed countries (2–13 eggs/g TS). According to Buitrón and Galván [16], it is essential to know if the remaining eggs after the treatment are viable because they pose a severe health risk. According to some sources, the sewage sludge also contains as many as 10^6^ microorganisms in 1 mL, and about 10% is pathogenic to animals and humans [33].

Contaminated environmental components thus can lead to the spread of microorganisms, which can further directly negatively impact human and animal health. The high tenacity of the endoparasitic stages in the outer environment compared to the other microorganisms increases the health risks. For example, pathogenic viruses and bacteria survive in the external environment for several hours to days, protozoan cysts for several months up to the year, while thick-shelled helminthic eggs may remain viable for several years due to the very high resistance of the eggs to the adverse environmental conditions [34,35,36,37,38]. The reason is that the cellular wall of eggs contains stabilizing proteins (keratin, elastin), lipids, and in the nematode eggs also chitin [39]. All these pathogens are potentially dangerous for public health because the infection dose is low, and in some parasites, it can be accounted for by only one egg [40,41,42]. Wang et al. [20] reported that conventional onsite wastewater treatment systems could potentially contribute to the transmission of infectious diseases caused by waterborne pathogenic microorganisms and become an essential human health concern. On the other hand, sewage sludge can be a valuable source of organic matter for agriculture as it contains functional agrochemical-nutrient components applicable to the soil. Sludge has a high amount of organically bound nitrogen and phosphorus [43,44]. However, it also contains components that may pose a risk to the soil, water, and the human food chain, particularly at higher concentrations. Thus, this material requires qualified processing and utilization to prevent soil contamination and avoid the accumulation of risk components in the soil.

In the Slovak Republic, the annual production of sludge reaches close to 340 thousand tons. This material is included within the category of special wastes [45], which must be safely disposed of mainly due to the presence of pathogens. On the other hand, sludge is a valuable source of nitrogen, phosphorus, potassium, and some trace elements.

This work aimed to study the effect of wastewater treatment on bacterial communities, antibiotic-resistant bacteria, and the occurrence of developmental stages of endoparasites.

## 2. Materials and Methods

### 2.1. Description of Investigated Wastewater Treatment Plants

Five wastewater treatment plants (WWTPs) located in central Slovakia were investigated to determine the effect of treatment on bacterial communities, antibiotic-resistant bacteria, and helminth eggs. This study was performed in five wastewater treatment plants that remain anonymous and are labeled as “WWTP A-E”. All wastewater treatment plants receive municipal wastewater and discharge the effluents into streams or rivers. The characteristics of studied wastewater treatment plants are described in Table 1. The generated sludge was treated by aerobic stabilization (WWTP B) or anaerobic stabilization (WWTP C) and applied to the compost. Sludge from WWTP A, D, and E was not treated in the plants. Unstabilized raw sludge in WWTP B was treated by aerobic stabilization. WWTP B was shut down during the monitored period, so obtaining samples from the influent portion was not possible.

### 2.2. Sample Collection

Samples were collected in the summer of 2020 (June–August). During the tested period, 9 samplings were performed and 3 samples were taken from each sampling place (influent, effluent, sludge).

Two liters of influent and effluent samples were collected to chemically clean bottles for the physicochemical evaluation, and 1 L was placed into a sterile bottle for microbiological and parasitological examination. A total of 2 L of raw sludge and 1 kg of treated sludge were collected. Samples were stored without any conservation at 4 °C and transferred to the laboratory for microbiological, parasitological, and chemical examination, performed within 24–48 h.

### 2.3. Chemical Examination of Samples

Chemical examination of wastewater (input and output) and sewage sludge included determination of pH, the levels of total nitrogen (N_t_), water-soluble ammonium (NH_4_-N), total phosphorus (P_t_), and chemical oxygen demand (COD_Mn_). According to STN ISO 10523 [46], the pH was determined with a pH-meter (HACH Company, Loveland, CO, USA) and a WATERPROOF pH Tester 30. COD_Mn_ was determined by oxidation with KMnO_4_ according to STN EN ISO 8467 [47] and NH_4_-N by titration [48,49]. A portion of samples for N_t_ determinations was digested using a HACH-Digesdahl apparatus (HACH Company, Loveland, CO, USA). N_t_ was distilled with NaOH (40%) [50,51], and P_t_ was determined by the vanadomolybdate method [52,53]. Efficiency of chemical pollutants was calculated according to the formula:

(1) $$\%\;\mathrm{of}\;\mathrm{efficiency}=\left(\frac{\mathrm{concentration}\;\mathrm{of}\;\mathrm{polutant}\;\mathrm{in}\;\mathrm{influent}-\mathrm{concentration}\;\mathrm{of}\;\mathrm{polutant}\;\mathrm{in}\;\mathrm{effluent}}{\mathrm{concentration}\;\mathrm{of}\;\mathrm{polutant}\;\mathrm{in}\;\mathrm{influent}}\right)\times 100\%$$

According to National Regulation SR [54], the biological process of wastewater treatment is focused only on the reduction of chemical pollutants, which have to meet the maximal limit of chemical factors in the effluent grab samples of wastewater discharged as described in Table 2.

### 2.4. Microbiological Examination of Samples

#### Isolation of Bacteria

The influent, effluent, and sludge wastewater samples were prepared by a serial dilution from 10^1^ to 10^6^. All samples were examined in duplicate. Determination of relevant bacterial counts (total count of bacteria, coliform bacteria, fecal coliform bacteria, and fecal enterococci) was carried out in compliance with the Slovak Republic Government Regulation [55]. The total bacterial count (TCB) was determined according to STN EN ISO 6222 [56]. The pour-plate method on meat-peptone agar was followed by aerobic cultivation at 37 °C for 24 h for bacteria determination.

Coliform bacteria (CB) and fecal coliform bacteria (FCB) were cultivated according to STN EN ISO 9308-1 [57] using Endo agar (HiMedia, Mumbai, India) and incubated for 24 h at 37 and 43 °C, respectively. Then, the characteristic colonies were counted. A lactose fermentation test confirmed coliform bacteria presence.

Determination of fecal enterococci counts (FE) was carried out according to STN EN ISO 7899-2 [58]. The cultivation of respective bacteria was performed on Slanetz Bartley agar (Merck, Darmstadt, Germany) at 37 °C for 48 h.

### 2.5. E. coli Isolation

The suspect *E. coli* colonies from Endoagar (or Chromagar, McConkey agar) were identified by biochemical ENTEROtest 24 (Erba Lachema, Brno, Czech Republic), intended to identify the critical species of the family Enterobacteriacea. Pure 24 h bacterial culture was inoculated in sterile saline solution. The homogenized suspension must have turbidity equal to No. 1 of the McFarland turbidity scale. The microtitration plates with 24 biochemical tests (urease, arginine, ornithine, lysine, hydrogen sulfide, simmons citrate, malonate, β-galactosidase, salicin, sorbitol, melibiose, cellobiose, lactose, trehalose, mannitol, β-glucuronidase, dulcitol, adonitol, arabitol, sucrose, inositol, raffinose, esculin, β-xylosidase) were inoculated with 0.1 mL of culture suspension. After 24 h of incubation at 37 °C, microtitration plates were analyzed for color reaction by reader ErbaScan and identification program ErbaExpert. The identification was supplemented by the paper strip tests: OXItest, COLItest, and PYRAtest (Erba Lachema, Brno, Czech Republic).

### 2.6. Determination of Minimal Antibiotics Inhibitory Concentrations in E. coli Isolates

A total of 142 isolates of *E. coli* (one sample, one strain) were analyzed for antibiotic susceptibility. Minimal inhibitory concentrations (MIC) were determined according to VET01-S2 [59] and EUCAST [60] by a Miditech system (Miditech, Bratislava, Slovakia) with the interpretative reading of MIC [61]. MIC GX expresses geometric mean MIC values for an antibiotic agent (mg/L) in *E. coli* isolates. The antibiotics used in the presented study were as follows: ampicillin (AMP), ampicillin and sulbactam (SAM), piperacillin + tazobactam (TZP), cefuroxime (CXM), cefotaxime (CTX), ceftazidime (CAZ), ceftazidime with clavulanic acid (CAC), cefoperazone and sulbactam (SPZ), cefepime (FEP), ertapenem (ETP), meropenem (MEM), gentamicin (GEN), tobramycin (TOB), amikacin (AMI), ciprofloxacin (CIP), tetracycline (TET), tigecycline (TGC), chloramphenicol (CHL), colistin (COL), trimethoprim and sulfonamides (COT) and nitrofurantoin (NIT). The antibiotic reading system automatically identifies common resistance mechanisms, such as “ESBL”, extended spectrum β-lactamases; “Multiresistance!“, current resistance in 3 or more unrelated ATB groups; “AAC(6′), AAC(3′), ANT(2′)”, enzyme-modifying aminoglycosides in G-bacteria; AGL AAC(6)—enzym AAC(6) modifying of aminoglycosides; “TEM-1, -2, SHV-1–low“, common beta-lactamases, with low enzyme expression; “TEM-1, -2, SHV-1–high“, common beta-lactamases with high enzyme expression; “Plasmidic AmpC!“, plasmid-transferred Amp C beta-lactamases; “Class A, Amp C, hyp.!“, derepressed chromosomal beta-lactamases!; “Carbapenem resistance“, resistance to carbapenems; Penicilinase high, penicilinase with high enzyme expression; Penicilinase low, penicilinase with low enzyme expression, etc.

### 2.7. Parasitological Examination of Samples

To determine protozoa (oo) cysts and helminth eggs count in influent and effluent samples from WWTP and sewage sludge, 50 mL from each 1 L sample was taken and examined by sedimentation and flotation techniques [62].

The dry sludge samples were surveyed according to Kazacos [63]. Briefly, 100 g of the pooled sludge sample, 100 mL of water, and 0.5 mL of Tween 40 were mixed and decanted for 10 min. Subsequently, the samples were sieved and replenished with 1000 mL of water. After 1 h of sedimentation, the soil samples were centrifuged (Eppendorf 5804, Hamburg, Germany) and then floated with sucrose flotation solution (specific density of 1.3). Samples were examined under the light microscope at 20× and 40× magnification (Leica Microsystems, DM 5000B light microscope, Wetzlar, Germany) to detect the presence of protozoa (oo) cysts and helminth eggs.

## 3. Results

WWTPs (A, D, E) are small, representing up to 10,000 residents. Here, the regularly monitored value is only one chemical indicator, i.e., CODMn (70 mg/L in grab samples or 135 mg/L in composite samples). In WWTP D and WWTP E, these values exceeded the norm (180 mg/L) and in WWTP E, this value exceeded the norm more than twice. Furthermore, in the biggest WWTP C, the CODMn was up to 282.8 mg/L, where only 125 mg/L is officially allowed. WWTP B was in reconstruction, and wastewater flowed directly to the river, and the COD level was exceeded twice Here the maximal limit was 140 mg/L (Table 3).

After mechanical and biological treatment in WWTPs, the effluent’s chemical factors decreased within the limit in every monitored wastewater treatment plant.

**Figure 1:**
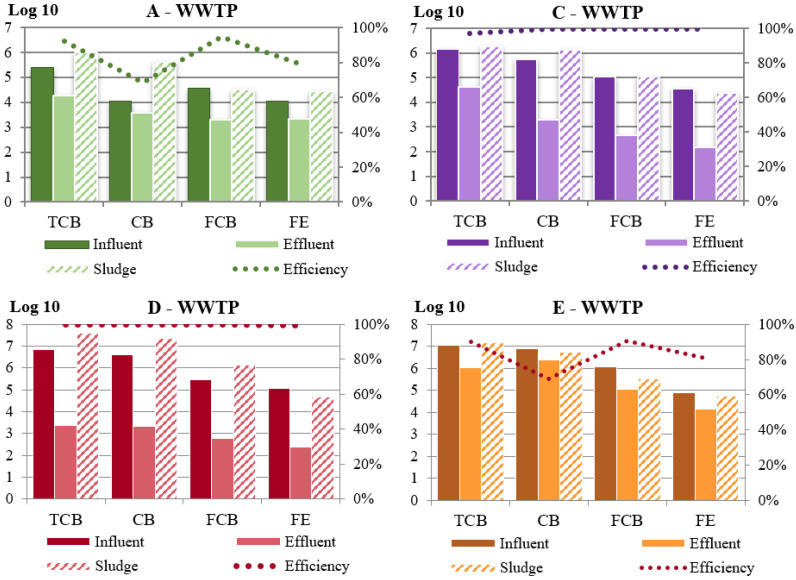
Comparison of bacterial contamination (log10) and treatment efficiency in four WWTPs (CB—coliform bacteria, FCB—fecal coliform bacteria, FE—fecal enterococci, TCB—total count bacteria).

The results of the microbial evaluation and the percentage of efficiency in the individual examined wastewater treatment plants are described in Figure 1. The log10 in the total number of microorganisms in individual wastewater treatment plants ranged from 7 to 5.4 in influent and from 6 to 3.4 in the effluent, representing a decrease from 99 to 89%. The total coliform bacteria concentration in the sludge varied from 7.6 to 5.7. The log10 of coliform bacteria ranged between 6.9 to 4 in the influent and in the effluent from 6.4 to 3.4, representing a 68 to 99% purification efficiency. The fecal coliform bacteria were 4.5 to 6 at the influent and from 2.6 to 4.9 in the effluent to express 95 to 99% efficiency. The log10 of fecal enterococci ranged from 4 to 4.4 in the influent and 2.2 to 4.2 in the effluent, thus showing the efficiency from 79 to 99%. The concentration of the monitored bacteria in the sludge was approximately the same as in the samples from the wastewater influent.

**Figure 2:**
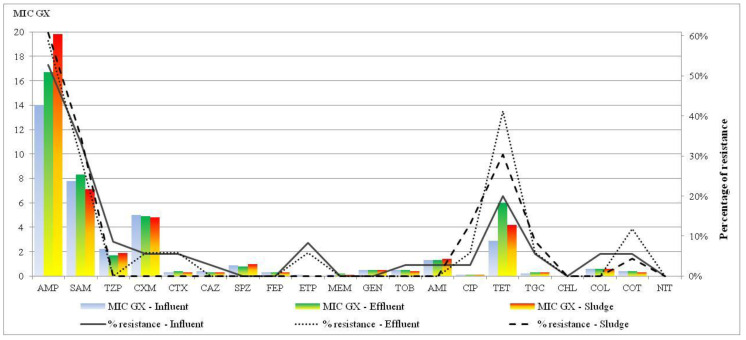
Comparison of MIC GX and percentages of resistance in influent, effluent, and sludge from the WWTPs.

A modified microdilution method with the VetMIC panel detected antimicrobial resistance in 142 *E. coli* strains. The highest incidence of beta-lactamase resistance was observed for ampicillin (60% and MIC GX 18.9 mg/L in sludge samples), which was followed by ampicillin and sulbactam (35.21% and MIC GX 7.8 mg/L), tetracycline (20% in influent and 40% in effluent, MIC GX 3.8 mg/L), piperacillin and tazobactam (8.6%, MIC GX 2.2 mg/L in influent). MICs of cefuroxime and cefotaxime were 5.8 in influent and 5.6 mg/L (in effluent); tigecycline, trimethoprim and sulfonamides was about 5.6% (in effluent). The percentage of resistance to ceftazidime, tobramycin, amikacin, ciprofloxacin was 2.87% in influent and effluent components, and sludge showed low resistance to those antibiotics. Resistance to meropenem, sulbactam, cefepime, meropenem, gentamicin, and nitrofurantoin was 0% in all wastewater and sludge samples (Figure 2).

**Figure 3:**
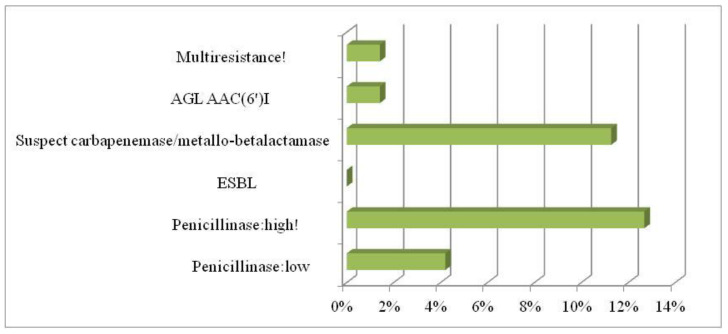
The phenotype of resistance in *E. coli* strains.

Among all 142 investigated *E. coli* strains, the high penicillinase phenotype was confirmed in 9 strains (12.68%) and 11.27% of carbapenemases (detected in two strains from the influent portion). Three phenotypes were penicillinase low (4.23%). An AGL AAC(6′) phenotype was also detected in samples. Multi-drug resistance was confirmed in one sample (Figure 3). Based on the MIC phenotypic identification of *E. coli* strains, 12.5% were high-level resistance penicillinase occurrence, 4% low-level, 1.4% were multiresistant isolates, and 1.4% were aminoglycosides AAC(6′)I. The ESBLs were not detected in isolated *E. coli* strains.

The results obtained from the parasitological examination are presented in Table 4. *Giardia duodenalis* was detected only in WWTP C influent section collected in June 2020. No other cysts were observed by microscopy in water or the sludge.

There were no helminth eggs found in effluent portions. *Ascaris* spp. eggs were confirmed in influent from WWTP A, B, and E. No *Ascaris* spp. eggs were detected in influent from WWTP C and D. It was impossible to differentiate between the species since only the optical microscopy detection was carried out. *Hymenolepis nana* eggs and eggs from the family Ancylostomatidae were also found in the influent portion. The most prevalent helminth eggs in sludge were *Ascaris* spp. eggs and eggs from the family Ancylostomatidae (Table 4).

## 4. Discussion

The treatment process in the WWTPs is primarily focused on the removal of chemical pollutants (COD, BOD5, N-NH4, total nitrogen, and total phosphorus) [54], which are checked in effluent values as a preventive measure against river pollution (eutrophication of water) negatively impacting river ecosystems [42,64].

During the studied period, almost all chemical factors in effluent from monitored WWTPs were in line with the standards and regulations for discharge into public waterways.

COD, the measure of the oxygen equivalent of the organic matter and microorganisms in the wastewater, was higher in smaller and bigger WWTPs and exceeded the maximum level of 125 mg/L. In untreated domestic wastewater, COD usually ranges between 250 and 1000 mg/L [65].

By comparing the results of chemical parameters and microbiological analyses in the effluent, the higher efficiency of wastewater treatment in WWTP A, C, and D is visible. Almost all monitored microorganisms here decreased by 2 to 3 logarithmic orders. The concentration of microorganisms in the sludge in individual WWTPs was comparable or slightly higher than at the influent. A comparison of the number of microorganisms in the sludge of the monitored WWTPs found no significant differences (ranging from 6–7 log in TCB, CB, and 4.5–6 log in FCB and FE).

The number of coliform bacteria in effluent ranged from 3.4 to 6.3 log CFU∙mL^−1^ which correlates with results of other works investigating wastewater in Slovak and Czech Republic (3.02–4.94 log CFU∙mL^−1^) [66]. In effluent wastewater from hospitals, the concentrations of a number of coliform bacteria can reach 7.18 log CFU∙mL^−1^ [67], which is in two orders higher when compared to the domestic and municipal WWTP, highlighting the importance of separated wastewater and sludge collection and treatment.

Interestingly, the effectiveness of the treatment process in small WWTP (up to 2000 recipient) is less than in big WWTP (up to 100,000 recipient). For example, in the WWTP E, the wastewater from 900 recipients had the highest concentration in chemical parameters and microbial concentration in influent decreased only by 0.5 log order. This signals low effectiveness of cleanability and possible failure in the treatment process. In addition, larger WTTPs employ more professional staff, the cleaning process is regularly controlled and inspected (including self-inspections), and the monitored parameters in the effluent are set up to be more strict.

Although the success of treatment by comparing the concentration of chemical parameters, microorganisms and parasites in effluent has been relatively high, it still poses a risk to rivers’ pollution and habitats. It is not proven that the treatment is sufficient to remove antibiotic-resistant bacteria. It is known that some of these bacteria can carry resistance genes either in their core genome or on mobile genetic elements. These genes can be transferred from one bacterium to another via horizontal gene transfer under selection pressure [68]. There are also possibilities for plasmid transfer in wastewater treatment plants and surface waters [69,70].

In a recent study, the following *E. coli* resistances were detected: more than 50% to ampicillin, 35% were resistant to ampicillin and sulbactam, 20% to tetracycline, and 8.6% to piperacillin and tazobactam. *E. coli* isolates from the effluent also showed resistance to cefotaxime, cefepime, cefuroxime, tigecycline, trimethoprim, and sulfonamides (around 5%). There were only minor differences between antibiotic resistance results from influent, effluent, and sludge.

We confirmed *E. coli* strains the Penicillinase: high, carbapenemase, Penicillinase: low, and AGL AAC(6′) phenotypes. A multidrug-resistant *E. coli* phenotype was detected in one sample.

In a previous study, Gregová and Kmeť [5] detected that the production of extended-spectrum β-lactamases in coliform isolates was encoded mainly by blaTEM, blaCTX-M-2, and blaCTX-M-8/25 genes. About 62% of resistant strains contained a combination of two or more extended-spectrum beta-lactamases (ESBL) genes.

Similarly, Reinthaler et al. [71] determined a 200-fold reduction of *E. coli* in WWTP effluent samples (from 10^4^ CFU·mL^−1^ to approx. 10^2^ CFU·mL^−1^). *E. coli* strains were resistant to the penicillin group (ampicillin 18%, piperacillin 12%), cephalosporin group (cephalothin 35%, cefuroxime 11%), quinolones (nalidixic acid 15%), trimethoprim/sulfamethoxazole (13%), and tetracycline (57%).

In untreated sludge, Redhead et al. [72] found higher resistance of *E. coli* isolates to amoxicillin, trimethoprim, ciprofloxacin, and gentamicin. After thermal hydrolysis at WWTPs, the absolute abundance was markedly reduced in *dfrA1*, *dfrA5*, *dfrA7*, *aac(3)-1*, CTX-M-1, CTX-M-9, *bla-Imp*, *qnrS*, *tetM*, *sul1*, and *intl1* genes, which is similar to the data observed by Martins et al. [73] and Raven et al. [74].

Lépesová et al. [67] have found that more than half of the coliform bacteria from hospital wastewater were multidrug resistant and possessed a strong biofilm-formation ability.

A high concentration of the monitored bacteria and antibiotic-resistant *E. coli* isolates was found in the sludge, which still poses a risk of spreading microorganisms through the direct incorporation of sludge into agricultural land. However, the above indicates that sludge produced during municipal wastewater treatment must be subjected to additional processing to improve its hygiene level. Therefore, composting constitutes an economically advantageous and hygienically effective method of sludge processing. In addition, processing and organic recycling wastes by composting must ensure a product that fulfills a range of water management and hygiene requirements and complies with the principles of waste legislation [45].

Still, wastewater treatment plants play a vital role in minimizing the discharge of many water pollutants, including antibiotics [75,76]. Degradation of hormones, pesticides, antibiotics, antihistamines, and drugs is limited, and as a result, they commonly end up in the aquatic environment. Runoff and wastewater discharges may also contribute to the spread of organic and inorganic nutrients that may boost the growth and proliferation of indigenous or introduced pathogens. 

*Ascaris* spp. eggs, viable eggs from the Ancylostomatidae family, and viable *H. nana* eggs were found in influent. This indicates that human feces and domestic animal excreta might contaminate wastewater treatment systems. During mechanical and biological treatment processes in the studied WWTPs, mainly bacteria, but not the endoparasites’ developmental stages, were devitalized. Therefore, all above-mentioned helminths egg sediment and concentrate were in the sludge with suspended particles. In the case of poor sludge treatment, they again re-enter the environment and pose potential hygiene, epidemiological or epizootiological risks [19,36,77]. Finally, we can state that the helminth eggs in the sludge indicate the overall health status and the incidence of diseases in the population monitored in the WWTP area.

An exciting finding was confirmation of *G. duodenalis* cysts influent. No cysts were found in the effluent stage and sludge. However, information about the presence of protozoan pathogens is limited due to the inconsistencies in sampling as well as concentration and recovery procedures [44]. Despite our findings, it is necessary to pay special attention to this parasite because it has great zoonotic potential. *G. duodenalis* (syn. *G. intestinalis*, *G. lamblia*) is a unicellular parasite causing gastrointestinal disorders in a wide range of hosts, including wild and companion animals as well as humans. It is one of the most spread parasitic diseases in man, who can become infected via the fecal–oral route through contaminated water (“water-borne”) and food (“food-borne”), or by direct transmission from host to host. This parasitic disease is classified as neglected and occurs more frequently in areas with poor hygiene standards, where children are the most affected population.

Similarly, Dudlová et al. [78] in east Slovakia studied the incidence of endoparasite germ’s developmental stages (cysts, oocysts, protozoa, and helminth eggs) as an indirect monitoring factor for endoparasite circulation in raw municipal wastewater, sludge, and biologically cleaned wastewater. The raw wastewater contained cysts of *Giardia* spp., and *Entamoeba* spp., the helminth eggs *Ascaris* spp., and strongyle-type eggs. No protozoa cysts or helminth eggs were found in the treated wastewater. The highest occurrence of endoparasite developmental stages was detected in drained stabilized sludge. Protozoan (oo) cysts (*Giardia* spp., *Cryptosporidium* spp., *Entamoeba* spp.) and helminth eggs (*Ascaris* spp., *Trichuris* spp., *Taenia* spp., *Hymenolepis* spp., or strongyle-type eggs) were found. In drained and stabilized sludge, the eggs of *Capillaria* spp. and *Toxocara* spp. were also detected. In comparison with our results, differences in parasite composition can be because wastewater treatment plants in eastern Slovakia are located close to territories with low hygiene standards and a significant presence of residents belonging to marginalized populations. On the other hand, Amoah et al. [79], Chaoua et al. [80], and others have reported that wastewater treatment plants are not fully capable of helminth eggs removal from the water.

We confirmed that sedimentation is an effective removal mechanism for the helminth eggs. Similar results are reported by Kansiime et al. [81] and Dai et al. [82]. In the low-quality water, the eggs are incorporated into particle flocs with different settling velocities, and the settling rate of eggs and particles is closely associated [83]. Environmental conditions can also affect the survival of the pathogen in wastewater. For example, the rate of removing bacterial indicators (e.g., *Salmonella* and fecal coliform bacteria) is higher in summer than in winter [84,85]. UV radiation by sunlight is an effective mechanism for pathogen removal, mainly in open-water treatment wetlands. Temperature, pH, insolation, and solar radiation influence the survival of *A. suum* eggs [86,87,88].

Based on our results, we can conclude that the use of untreated or insufficiently treated wastewater poses many risks. The use of untreated wastewater is also associated with a higher infection intensity, especially for helminth infection. Ensink et al. [89] found a significantly increased risk of *A. lumbricoides*, hookworm, and *T. trichiura* infection in farming communities irrigating with wastewater. Aquatic recipients contaminated with wastewater containing helminth eggs also have a high concentration of suspended particles resulting in flocculation of the suspended material, including eggs [83].

## 5. Conclusions

Wastewater treatment plants play a vital role in minimizing the discharge of many water pollutants and the protection of the environment. The results show that the pattern of resistance in *E. coli* isolates found in monitored WWTPs is comparable with resistance seen at the clinical health care level. Ampicillin (beta-lactams), gentamicin (aminoglycoside), and ciprofloxacin (fluoroquinolones) are frequently prescribed drugs in Slovakia.

However, the main wastewater and sludge reuse limitation is contamination with helminth eggs due to its low infective dose and prolonged survival in the environment. In this study, several parasite species were identified in influent segments and sludge, where nematodes of human and animal origin were predominantly identified. In addition, the wastewater and sludge indicates potential health risks for humans and workers at these sites.

## Figures and Tables

**Table 1:** Characteristics of the wastewater treatment plants studied.

	WWTP				
	A	B	C	D	E
Number of residents	630	7529	87,126	454	881
Discharge (m^3^/day)	550.8	3299.6	24,698.1	112.1	73.9
Discharge (l/s)	6.38	38.06	283.41	1.29	0.86
Recipient river	Turiec	Teplica	Váh	Vríca	Blatnický potok
Cleaning method	Mechanical and biological				
Treatment of sludge	Unstabilized sludge (treatment at WWTP B)	Aerobic stabilization, sludge applied to the compost	Anaerobic stabilization, sludge applied to the compost	Unstabilized sludge (treatment together WWTP B)	Unstabilized sludge (treatment at WWTP B)
Sewage type	Domestic	Domestic	Domestic	Domestic	Domestic

WWTP—wastewater treatment plant.

**Table 2:** Maximal limit of chemical factors in the effluent grab samples of wastewater discharged to the recipient rivers [54].

Number of Recipient	N_t_ (mg/L)	NH_4_-N (mg/L)	P_t_ (mg/L)	COD_Mn_ (mg/L)	IS (mg/L)
up to 50	−	−	−	−	−
51–2000	−	−	−	170	60
2001–10,000	−	40	−	170	50
10,001–25,000	40	30	−	140	50
25,001–100,000	30	20	5	125	40
over 100,000	25	10	4	125	40

N_t_—total nitrogen; NH_4_-N—water-soluble ammonium nitrogen; P_t_—total phosphorus; COD_Mn_—chemical oxygen demand; IS—insoluble substances; − means no maximal limit.

**Table 3:** Physico-chemical analysis of wastewater before and after treatment in WWTPs.

	N_t_ (mg/L)	NH_4_-N (mg/L)	P_t_ (mg/L)	COD_Mn_ (mg/L)	pH
WWTP A					
Influent	22.41	14.01	17.02	214.84	7.13
Effluent	14.01	3.50	1.05	152.65	7.01
Sludge	249.33	11.21	32.76	290.30	6.70
Efficiency	37.5%	75.0%	93.8%	28.9%	
WWTP B					
Influent	44.83	15.41	26.37	478.40	7.30
WWTP C					
Influent	39.22	51.83	67.82	537.35	7.45
Effluent	12.61	3.50	2.71	282.80	7.34
Sludge	424.41	11.80	129.65	2467.475	6.78
Efficiency	67.9%	93.2%	96.0%	47.4%	
WWTP D					
Influent	39.22	26.61	108.14	348.95	7.26
Effluent	18.21	4.90	2.57	180.41	7.60
Sludge	260.53	21.01	86.20	863.47	6.92
Efficiency	53.6%	81.6%	14.4%	48.3%	
WWTP E					
Influent	103.65	81.24	133.96	936.90	7.93
Effluent	82.64	35.02	5.36	414.83	7.16
Sludge	140.07	43.42	71.79	623.22	6.95
Efficiency	20.3%	56.9%	96.0%	55.7%	

WWTP—wastewater treatment plant; N_t_—total nitrogen; NH_4_-N—water-soluble ammonium nitrogen; P_t_—total phosphorus; COD_Mn_—chemical oxygen demand; IS—insoluble substances.

**Table 4:** Parasitological examination of wastewater before and after treatment in WWTPs.

WWTP	Sample	Eggs/Oocysts
WWTP A	Influent	*Ascaris* spp., *Hymenolepis nana*, family Ancylostomatidae
	Effluent	Neg.
	Sludge	*Ascaris* spp., family Ancylostomatidae
WWTP B		
	Influent	*Hymenolepis nana*, *Ascaris* spp., family Ancylostomatidae

WWTP C	Influent	*Hymenolepis nana*, *Giardia duodenalis*
	Effluent	Neg.
	Sludge	*Ascaris* spp., family Ancylostomatidae
WWTP D	Influent	Neg.
	Effluent	Neg.
	Sludge	*Ascaris* spp., family Ancylostomatidae
WWTP E	Influent	*Ascaris* spp.
	Effluent	Neg.
	Sludge	family Ancylostomatidae

## Data Availability

Data are available from the corresponding author upon reasonable request.
